# Supreme Court State of New York

**Published:** 1904-05

**Authors:** Thomas L. Hamilton

**Affiliations:** Clerk


### SUPREME COURT STATE OF NEW YORK.

### COUNTY OF NEW YORK.

Fairchild Brothers & Foster,
a corporation, Plaintiff,
against
Morris Dlugach,
and
Herman Finkelstein,
doing business under the firm name or style of Broadway Drug Company, Defendants.

The summons in this action, dated the 20th day of October, 1903, and the complaint herein, verified the 20th day of October, 1903, having been duly served on the defendants on the 21st day of October, 1903, together with an order to show cause, containing a preliminary injunction against the defendants and each of them, dated the 21st day of October, 1903, and an undertaking having been filed by the plaintiff herein and duly approved by the Court, and an order of injunction *pendente lite* having been granted and entered herein on the 30th day of November, 1903; and the defendants having answered by their answer verified the 9th day of November, 1903, and having on the 23d day of March, 1904, offered in writing to allow judgment to be taken against them to the effect that "the said defendants and each of them, and their servants, agents and employees, and all persons acting in their behalf, be prohibited, restrained and enjoined perpetually from selling, dispensing, advertising or displaying at the drug-store of said defendants at No. 177 Broadway, Borough of Manhattan, City of New York, or elsewhere, any chemical or pharmaceutical preparations of any sort or kind whatsoever bearing signs, labels or wrappers marked 'Fairchild' or 'Dr. Fairchild', or any similar word or words, or purporting to be by 'Dr. Fairchild' or 'Fairchild', which said preparations are not manufactured by plaintiff"; and the plaintiff, on the 23d day of March, 1904, the same being within ten (10) days after service of said offer of judgment, having accepted said offer, as appears by the affidavit of Arthur F. Gotthold, duly verified the 23d day of March, 1904, and hereto annexed; and the parties herein having adjusted the money damages and costs as prayed for in the complaint;

Now on motion of Gould & Wilkie, attorneys for the plaintiff herein, it is

ADJUDGED that the defendants and each of them and their servants, agents and employees, and all persons acting in their behalf, be and they are hereby prohibited, restrained and enjoined perpetually from selling, dispensing, advertising or displaying at the drug-store of said defendants at No. 177 Broadway, Borough of Manhattan, City of New York, or elsewhere, any chemical or pharmaceutical preparations of any sort or kind whatsoever bearing signs, labels or wrappers marked "Fairchild" or "Dr. Fairchild", or any similar word or words, or purporting to be made by "Dr. Fairchild" or "Fairchild", which said preparations are not manufactured by plaintiff.

Dated, March 24th, 1904. THOMAS L. HAMILTON,
Clerk.

